# Tuberculosis treatment outcome and its associated factors among people living with HIV and AIDS in Fako Division of Cameroon

**DOI:** 10.1371/journal.pone.0218800

**Published:** 2019-07-30

**Authors:** Elvis Asangbeng Tanue, Dickson Shey Nsagha, Theophile Nana Njamen, Nguedia Jules Clement Assob

**Affiliations:** 1 Department of Public Health and Hygiene, Faculty of Health Sciences, University of Buea, Buea, Cameroon; 2 Department of Obstetrics and Gynecology, Faculty of Health Sciences, University of Buea, Buea, Cameroon; 3 Department of Medical Laboratory Sciences, Faculty of Health Sciences, University of Buea, Buea, Cameroon; University of South Carolina Arnold School of Public Health, UNITED STATES

## Abstract

**Background:**

Tuberculosis (TB) and HIV co-infection challenges treatment and worsens the outcome of TB treatment. This study aimed to assess the outcome of TB treatment and factors facilitating treatment success among people living with HIV/AIDS in Fako Division of the South West Region of Cameroon.

**Methods:**

A hospital-based retrospective cohort study was conducted by manually reviewing medical records of HIV/TB co-infected patients from January 2010 to September 2017. A structured data collection form was used to review the medical records of HIV patients co-infected with TB aged 10 years and older. Patients with incomplete files were dropped from the study. Treatment success was defined as the sum of patients who were declared cured and those who had completed treatment, as per the World Health Organization’s recommendations. Data were analyzed using Statistical Package for Social Sciences version 21. Bivariate and multivariate logistic regression model was carried out to identify factors facilitating successful TB treatment outcome. Significance was obtained through adjusted odds ratio with its 95% confidence interval and a p<0.05.

**Results:**

A total of 2,986 files were reviewed but 2,928 (98.1%) were retained. Out of the 2,928 medical files of adult TB patients reviewed, 1,041 (35.6%, [95% CI 33.8%-37.3%]) were HIV/TB co-infected. The 1,041 co-infected patients had a mean age of 37.07 (SD of10.02) years and 56.3% were females. The treatment outcome of TB patients were 795(76.4%) cured, 23(2.2%) treatment completed, 99(9.5%) were lost to follow-up, 16 (1.5%) failed, 72(6.9%) died and 36(3.5%) transferred out. A successful treatment outcome was achieved in 818(78.6%,[95% CI: 76.0%–81.0%]) patients. Being a female [COR 1.61, 95% CI: 1.19–2.17, p = 0.002], receiving TB treatment in 2014 [COR 2.00, 95% CI: 1.11–3.60, p = 0.021] and 2015 [COR 2.50, 95% CI: 1.39–4.50, p = 0.002], having relapsed TB infection [COR 0.46, 95% CI: 0.23–0.93, p = 0.031], receiving ART [COR 1.95, 95% CI: 1.28–2.97, p = 0.002] and Cotrimoxazole [COR 2.03, 95% CI: 1.12–3.66, p = 0.019] were factors significantly associated with successful treatment. After adjusting for confounders, successful treatment outcome were associated with being a female [AOR 1.6; 95% CI: 1.21–2.22, p = 0.001], diagnosis of TB in 2014 [AOR 1.90; 95% CI: 1.04–3.45, p = 0.036] and 2015 [AOR 2.43; 95% CI: 1.33–4.43, p = 0.004].

**Conclusion:**

There is a high TB treatment success rate among HIV/TB co-infected patients in our setting, although below the target set by the WHO. Specific interventions aimed at enhancing patient outcomes are recommended.

## Background

Tuberculosis (TB) remains a major public health problem especially in resource-poor settings [1]. Globally, TB is the largest cause of death among people living with HIV/AIDS (PLWHA) [1]. The WHO Global TB Report of 2018 estimated 10.0 million new cases of TB occurred in 2017, of which over 82% of TB deaths occurred in Low and Middle-Income Countries. In 2017, there were an estimated 0.9 million new cases of TB amongst PLWHA, 72% of whom were living in Africa [1]. TB was a leading killer of PLWHA in 2017, with 0.3 million of TB related death [1]. Cameroon has been classified among the 30 high burden countries with TB and HIV/AIDS infections in the world [1].

HIV/AIDS predisposes for *Mycobacterium tuberculosis* (MTB) infection and increases the rate of recurrent TB as well as of rapid progress to active TB among individuals with recently acquired infection [2]. The risk of progressing from latent to active TB is estimated to be about 20 times greater in PLWHA than among those without HIV infection with a higher risk of transmitting the infection to others [3].

Tuberculosis and HIV co-infection is associated with significantly increased likelihood of mortality with HIV co-infected TB patients having significantly lower cure rates and lower treatment success rates compared to non-HIV infected TB patients [4]. The incidence of TB in Cameroon was 194 cases per 100,000 populations in 2017 [1]. In 2017, the incident rate of TB was 60 cases per 100,000 among PLWHA [1]. This indicates a considerable co-infection rate from both diseases. However, there is lack of evidence on TB treatment outcomes among co-infected patients in Cameroon. There is no readily available data describing TB treatment outcomes among PLWHA in the South West Region of Cameroon. An understanding of TB treatment outcomes and associated factors may help to improve in the management of the TB infection. Therefore, this study assessed TB treatment outcomes and its associated factors among PLWHA in the Fako Division of the South West Region of Cameroon.

## Methods

### Study area

The study was conducted in some health facilities in the Fako Division, South West Region of Cameroon; they included the TB units of the Regional Hospital Limbe, Regional Hospital Buea, Muyuka District Hospital and Tiko Central Clinic. These health facilities serve the population of the region and its environs. The region is characterized by two main seasons; the rainy season, which lasts from March to October, and the dry season, which lasts from November to February. The mean temperature ranges from 2°C in the coldest months to 33 °C in the hottest months. The population is cosmopolitan, with many ethnic groups attracted to the division by its fertile volcanic soil suitable for farming activities.

### Study design and setting

A retrospective cohort study was conducted by reviewing on a 93-month period (January 2010 to September 2017), medical records of HIV and TB infected patients. The data extraction took place over a period of four months in all the facilities (March to June, 2018).

### Target population: Inclusion and exclusion criteria

The study targeted HIV patients diagnosed with active TB disease. The accessible population was all PLWHA diagnosed with active TB disease and put on anti-TB treatment regimens in the selected health facilities during the period extending from January 2010 to September 2017. Patients with age ≥10 years who had started and completed a course of anti-TB therapy were included in the study. Participants’ medical records were reviewed to extract socio-demographic details of patients, TB type, medications and treatment outcomes. The exclusion criteria of the study were files of patients with incomplete medical records (unknown HIV status and unknown treatment outcome).

### Data collection

A structured data collection form that contained information on socio-demographic characteristics, medication-related factors and treatment outcome was prepared and used to extract the data from patients’ medical records. The data source for the study was the TB register at the TB clinics of the health facilities where all patients diagnosed with active TB disease are put on anti-TB treatment regimens and monitored throughout the course of the treatment. The TB registers in the TB clinics of these health facilities were used to record all relevant information for the treatment and monitoring of TB patients. Data was extracted from different medical files, arranged monthly and yearly. The data search was carried out manually. The data collected was in four parts. The first section focused on the demographic characteristics of patients including sex, age and year of diagnosis. The second section focused on the clinical characteristics of patients such as TB disease category, antiretroviral and cotrimoxazole treatments and phase of TB treatment. The third section recorded data on the laboratory characteristics of patients such as the TB bacilli burden at each follow-up diagnosis and the final TB diagnosis. The fourth section was based on final TB treatment outcome whether the patients were cured or completed treatment or lost to follow-up and if they were transferred out or dead. Files of patients with incomplete information were dropped from the review.

### Operational definitions of key terms

**TB infection:** Infection with the bacilli of *Mycobacterium tuberculosis*.

**Active TB disease:** Presence of signs and symptoms of TB disease in an individual who is infected with the bacilli of *Mycobacterium tuberculosis*.

**Case of tuberculosis:** A definite case of pulmonary TB with one or more initial sputum smear positive for acid-fast bacilli or one in which a health worker has diagnosed TB and has decided to treat the patient with a full course of DOTs.

**HIV infection:** Infection with the Human Immune-deficiency Virus (HIV) that is confirmed by first and second line serologic tests.

**HIV/TB co-infection:** The presence of both HIV and TB infection in an individual patient.

**TB treatment outcome:** The final known status of a TB patient who was started on anti-TB treatment.

**Cured:** An initially sputum smear-positive patient who is sputum smear negative at or one month prior to, the completion of TB treatment and on at least one previous occasion (usually at the end of the second or fifth month).

**Treatment completed:** A patient who completed anti-TB treatment without evidence of failure but for whom sputum smear or culture results are not available in the last month of treatment and on at least one previous occasion.

**Treatment failure:** A patient whose sputum smear or culture is positive at the fifth monthof treatment or later during the course of treatment. Also included in this definition are patients found to harbor a multi-drugresistant strain at any point of time during the treatment, whether they are smear-negative or positive.

**Lost to follow-up:** A patient who has been on treatment for at least four weeks and whose treatment was interrupted for eightweeks.

**Died:** A patient who died for any reason during the course of treatment.

**Transfer out:** A patient who started treatment and was transferred to another treatmentunit and for whom the treatment outcome is not known at the time of evaluation of treatment results.

**Treatment success:** The sum of patients who were declared ‘cured’ and those who had ‘completed’ treatment.

### Data quality

Data extraction form was pre-tested at a health facility providing similar services to patients prior to the commencement of the actual data collection. Data were collected by trained research assistants with close supervision and assistant of the principal investigator. Completeness of the data was checked, coded and entered into the computer using the Statistical Package for Social Sciences (SPSS) version 21 software. Each entry was cross checked independently to ensure the quality of data.

### Ethical considerations

Ethical approval was obtained from the Institutional Review Board of the Faculty of Health Sciences of the University of Buea (Reference Number: 2018/147/UB/SG/IRB/FHS). Administrative authorization was obtained from the South West Regional Delegation of the Ministry of Public Health and the District Health Services and from the directors of the hospitals. Confidentiality of the participants’ information was maintained by giving participant’s code number and the data secured in the cupboard and computer with password. All data for the study were routinely collected as part of patient treatment and care, and were fully anonymous before we accessed them. The IRB approved all aspects of the study protocol.

### Data management and statistical analysis

Data obtained from each participant was entered into the research log book and checked by the lead author. The data was later keyed into a computer using Microsoft excel, 2016 (Microsoft Corporation Inc. USA) and verified for the possibility of entering errors. Descriptive statistics were utilized to summarize patients’ characteristics across the outcome variables. Associations between successful TB treatment outcomes and independent variables were assessed using the bivariate and multivariate logistic regression models. The outcome was categorized as successful (cured and treatment completed) and unsuccessful (lost to follow-up, failure, death and transferred out) treatment outcomes. Data were analyzed using SPSS version 21. Statistical significance was obtained through adjusted odds ratio with its 95% confidence interval and p<0.05.

## Results

### Demographic characteristics of HIV patients Co-infected with TB

A total of 2,986 files were reviewed but 2,928 (98.1%) met the inclusion criteria. The medical records of 2,928 adult TB patients were reviewed at the TB units of four hospitals in Fako Division from January 1, 2010 to September 31, 2017, of which 1,041 were HIV/TB co-infected. The female gender dominated the study with 56.3% rate of HIV/TB co-infection. The mean (±SD) age of participants was 37.07(±10.02) years with, 438(42.1%) of the participants being of the 31 to 40 years age group. Majority, 658(63.2%) of the co-infections were diagnosed during the Cameroon national strategic TB treatment plan of 2010 to 2014. The baseline demographic characteristics of study participants are summarized in Table 1.

**Table 1:** Demographic characteristics of HIV patients co-infected with TB in Fako Division from 2010 to 2017.

Variables	Category	Frequency (%)
**Sex**	Male	450(43.2)
	Female	591(56.3)
	Total	1,041(100.0)
**Age group**	Mean±SD	37.07±10.02
	<21 years	19(1.8)
	21–30 years	262(25.2)
	31–40 years	438(42.1)
	41–50 years	227(21.8)
	51–60 years	69(6.6)
	>60 years	26(2.5)
	Total	1,041(100.0)
**Treatment centre**	Regional Hospital Limbe	507(48.7)
	Regional Hospital Buea	234(22.5)
	Muyuka District Hospital	164(15.8)
	Tiko Central Clinic	136(13.1)
	Total	1,041(100.0)
**Year of TB diagnosis**	2010	123(11.8)
	2011	127(12.2)
	2012	112(10.8)
	2013	156(15.0)
	2014	140(13.4)
	2015	162(15.6)
	2016	134(12.9)
	2017	87(8.4)
	Total	1,041(100.0)
**TB strategic plan**	Within Strategic Plan	658(63.2)
	Post Strategic Plan	383(36.8)
	Total	1,041(100.0)

### Clinical characteristics of HIV patients co-infected with TB

Out of 1,041 participants, the majority [938 (90.1%)] presented with new diagnosis for TB disease. Of the total PLWHA, 925(88.9%) were on antiretroviral therapy whereas, 989(95.0%) were on Cotrimoxazole prophylaxis. Most (800(76.8%)) of the co-infected patients completed their DOTs programme. The baseline clinical characteristics of study participants are summarized in Table 2.

**Table 2:** Clinical characteristics of HIV patients co-infected with TB in the Fako Division from 2010 to 2017.

Variables	Category	Frequency (%)
TB category	New	938(90.1)
	Relapse	36(3.5)
	Treatment failure	8(0.8)
	Retreatment	14(1.3)
	Transferred in	45(4.3)
	Total	1,041(100.0)
ART regimen at TB diagnosis	Yes	925(88.9)
	No	116(11.1)
	Total	1,041(100.0)
Cotrimoxazole treatment at TB diagnosis	Yes	989(95.0)
	No	52(5.0)
	Total	1,041(100.0)
Phase of treatment at occurrence of TB outcome	No DOT	24(2.3)
	Intensive phase of DOT	179(17.2)
	Continuation phase of DOT	38(3.7)
	DOT full course	800(76.8)
	Total	1,041(100.0)
Number of follow-up diagnosis	One	143(13.7)
	Two	62(6.0)
	Three	37(3.6)
	Four	799(76.8)
	Total	1,041(100.0)

### Laboratory characteristics of HIV Patients co-infected with TB

Of the total PLWHA involved in the study, 373(35.8%) had a light (+) acid fact bacilli burden at diagnostic microscopy whereas only 6(0.6%) had a heavy (++++) burden. Most (872(83.8%)) of the PLWHA included in the study had a smear negative status at the assessment of their TB outcome (Table 3).

**Table 3:** Laboratory characteristics of HIV patients co-infected with TB in the Fako Division from 2010 to 2017.

Variables	Category	Frequency (%)
TB burden at diagnostic microscopy	+	373(35.8)
	++	281(27.0)
	+++	381(36.6)
	++++	6(0.6)
TB burden at first follow-up microscopy	+	58(59.5)
	++	21(21.6)
	+++	15(15.5)
	++++	3(3.1)
TB burden at second follow-up microscopy	+	8(57.1)
	++	2(14.3)
	+++	4(28.6)
TB burden at third follow-up microscopy	+	6(75.0)
	++	2(25.0)
Final TB diagnosis	Smear negative	872(83.8)
	Smear positive	169(16.2)

### Prevalence of HIV/TB co-infected among tuberculosis patients in the Fako Division from 2010 to 2017

**Fig 1:**
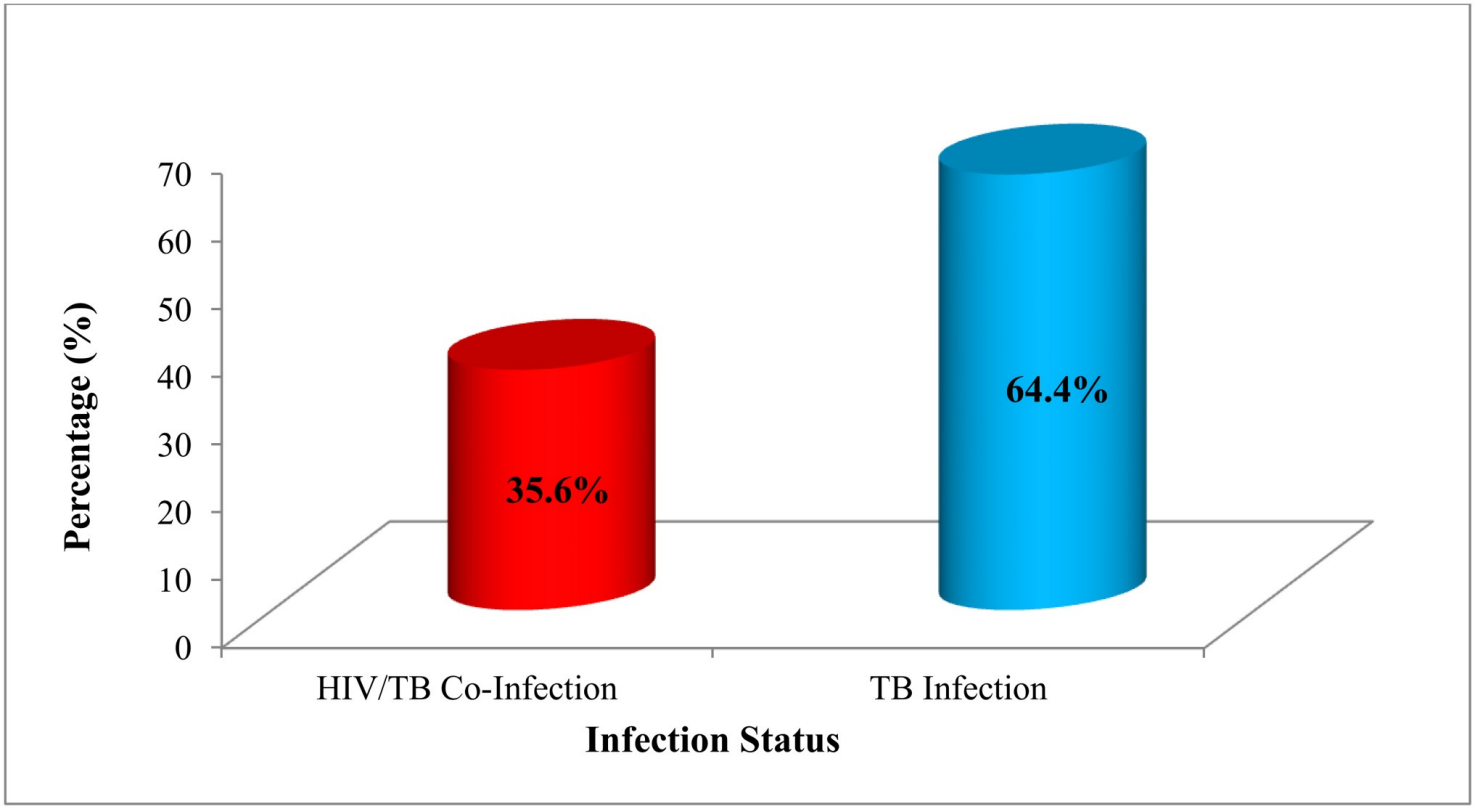
Prevalence of HIV/TB co-infection in the Fako Division from 2010 to 2017.

**Fig 2:**
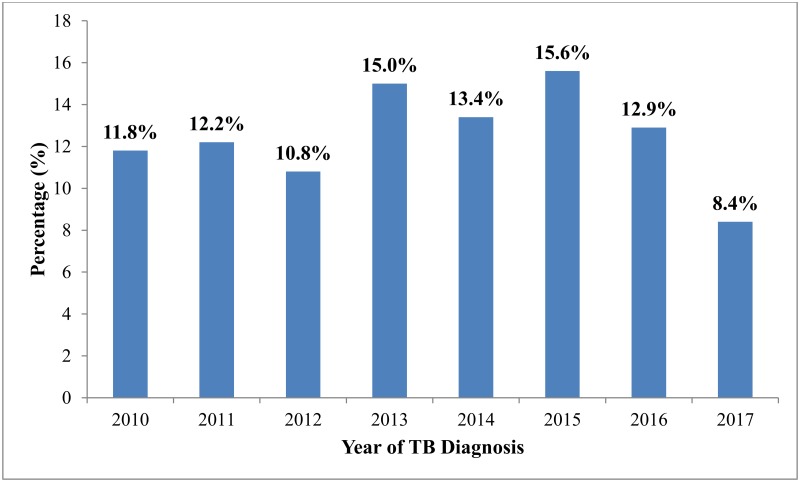
Yearly prevalence of HIV/TB co-infection in the Fako Division from 2010 to 2017.

The prevalence of HIV among TB patients was 35.6% [95% CI: 33.8%-37.3%] (Fig 1). The years 2013 and 2015 witnessed the highest rates of HIV/TB co-infection, 15.0% and 15.6% respectively (Fig 2).

### TB treatment outcome among HIV/TB co-infected patients in the Fako Division from 2010 to 2017

**Fig 3:**
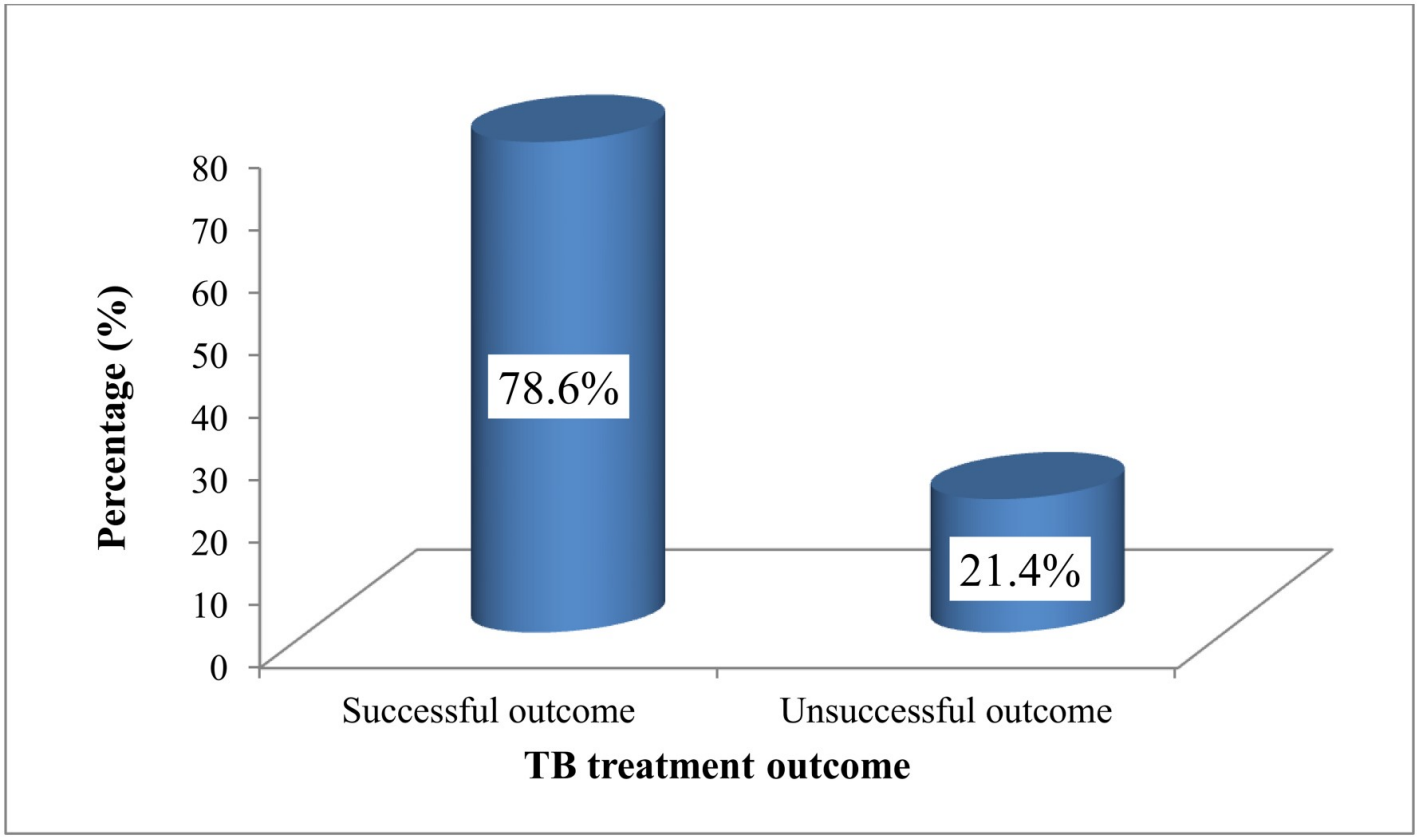
Success of TB treatment among HIV/TB co-infected patients in the Fako Division from 2010 to 2017.

As shown in Fig 3, of the 1,041 HIV/TB co-infected patients who were included in this study, 818 (78.6%) (95% CI:76.0%–81.0%) had successful TB treatment outcome. Majority, 795 (76.4%) of the participants were declared “cured” of TB co-infection while a handful of these patients, 72(6.9%) died in the course of the treatment (Table 4). The ability to complete treatment was similar between males and females (Table 5).

**Table 4:** TB treatment outcome among HIV/TB co-infected patients in the Fako Division from 2010 to 2017.

TB treatment outcome	Category	No (%)
Successful outcome	Cured	795(76.4)
	Treatment completed	23(2.2)
Unsuccessful outcome	Lost to follow-up	99(9.5)
	Failure	16(1.5)
	Died	72(6.9)
	Transferred out	36(3.5)

**Table 5:** Success of TB treatment by gender among HIV/TB co-infected patients in the Fako Division from 2010 to 2017.

		Successful TB treatment Outcome		
		Cured n(%)	Completed treatment n(%)	Total n(%)
**Gender**	**Male**	322(96.7)	11(3.3)	333(100.0)
	**Female**	473(97.5)	12(2.5)	485(100.0)

χ^2^ = 0.497, p = 0.481

### Factors associated with successful TB treatment outcome among HIV/TB co-infected patients

Table 6 provides a detailed account of the results of the binary analysis of factors associated with TB treatment outcomes. Female patients (COR 1.61, 95% CI: 1.19–2.17, p = 0.002) were significantly associated with successful treatment outcome as compared with males. Patients who were receiving TB treatment in 2014 (COR 2.00, 95% CI: 1.11–3.60, p = 0.021) and 2015 (COR 2.50, 95% CI: 1.39–4.50, p = 0.002) were significantly associated with successful treatment outcome as compared with those of 2010. The odds of having successful TB treatment outcome was 54% lower among patients with relapsed TB infection as compared to those newly diagnosed with TB (COR 0.46, 95% CI: 0.23–0.93, p = 0.031). Patients who were receiving ART (COR 1.95, 95% CI: 1.28–2.97, p = 0.002) and Cotrimoxazole (COR 2.03, 95% CI: 1.12–3.66, p = 0.019) at the time of TB diagnosis were significantly associated with successful treatment outcome compared to those who were not receiving these treatments.

**Table 6:** Binary regression analysis of factors associated with successful TB treatment outcome among HIV/TB co-infected patients in the Fako Division from 2010 to 2017.

Variables	Category	TB treatment outcome		Total	COR	95% CI	p-value
		Unsuccessful No (%)	Successful No (%)				
**Sex**	Male	117(26.0)	333(74.0)	450	1.00	-	-
	Female	106(17.9)	485(82.1)	591	1.61	1.19–2.17	0.002
**Age group**	<21 years	5(26.3)	14(73.7)	19	1.48	0.40–5.45	0.554
	21–30 years	62(23.7)	200(76.3)	262	1.71	0.73–4.02	0.221
	31–40 years	93(21.2)	345(78.8)	438	1.96	0.85–4.55	0.115
	41–50 years	41(18.1)	186(81.9)	227	2.40	1.00–5.77	0.050
	51–60 years	13(18.8)	56(81.2)	69	2.28	0.83–6.25	0.109
	>60 years	9(34.6)	17(65.4)	26	1.00	-	-
**Treatment centre**	RHL	101(19.9)	406(80.1)	507	1.04	0.65–1.67	0.863
	RHB	45(19.2)	189(80.8)	234	1.09	0.64–1.86	0.752
	MDH	49(29.9)	115(70.1)	164	0.61	0.36–1.04	0.068
	TCC	28(20.6)	108(79.4)	136	1.00	-	-
**Year of TB diagnosis**	2010	36(29.3)	87(70.7)	123	1.00	-	-
	2011	33(26.0)	94(74.0)	127	1.18	0.68–2.05	0.562
	2012	26(23.2)	86(76.8)	112	1.37	0.76–2.46	0.294
	2013	34(21.8)	122(78.2)	156	1.49	0.86–2.56	0.154
	2014	24(17.1)	116(82.9)	140	2.00	1.11–3.60	0.021
	2015	23(14.2)	139(85.8)	162	2.50	1.39–4.50	0.002
	2016	28(20.9)	106(79.1)	134	1.57	0.89–2.77	0.122
	2017	19(21.8)	68(78.2)	87	1.48	0.78–2.81	0.229
**Era**	Within SP	153(23.3)	505(76.7)	658	1.00	-	-
	Post SP	70(18.3)	313(81.7)	383	1.36	0.99–1.86	0.060
TB category	New	195(20.8)	743(79.2)	938	1.00	-	-
	Relapse	13(36.1)	23(63.9)	36	0.46	0.23–0.93	0.031
	Treatment failure	1(12.5)	7(87.5)	8	1.84	0.23–15.02	0.570
	Retreatment	4(28.6)	10(71.4)	14	0.66	0.20–2.11	0.480
	Transferred in	10(22.2)	35(77.8)	45	0.92	0.45–1.89	0.817
ART regimen at TB diagnosis	Yes	185(20.0)	740(80.0)	925	1.95	1.28–2.97	0.002
	No	38(32.8)	78(67.2)	116	1.00	-	-
Cotrimoxazole treatment at TB diagnosis	Yes	205(20.7)	784(79.3)	989	2.03	1.12–3.66	0.019
	No	18(34.6)	34(65.4)	52	1.00	-	-
TB burden at diagnostic microscopy	+	80(21.4)	293(78.6)	373	0.73	0.08–6.36	0.778
	++	70(24.9)	211(75.1)	281	0.60	0.07–5.25	0.647
	+++	72(18.9)	309(81.1)	381	0.86	0.10–7.46	0.890
	++++	1(16.7)	5(83.3)	6	1.00	-	-
TB burden at first follow-up microscopy	+	11(19.0)	47(81.0)	58	2.14	0.18–25.7	0.550
	++	3(14.3)	18(85.7)	21	3.00	0.20–44.36	0.424
	+++	3(20.0)	12(80.0)	15	2.00	0.13–30.16	0.617
	++++	1(33.3)	2(66.7)	3	1.00	-	-

COR = Crude Odds Ratio; CI = Confidence Interval; RHL = Regional Hospital Limbe; BRH = Buea Regional Hospital; MDH = Muyuka District Hospital; TCC = Tiko Central Clinic; SP = Strategic Period

In the multivariate analysis, being a female (AOR 1.6; 95% CI:1.21–2.22, p = 0.001), diagnosed with TB in 2014 (AOR 1.90; 95% CI:1.04–3.45, p = 0.036) and 2015 (AOR 2.43; 95% CI:1.33–4.43, p = 0.004) were significant determinants of successful TB treatment outcome among PLWHA. The odds of having successful TB treatment outcome remained lower (52%) among patients with relapsed TB infection (AOR 0.48, 95% CI: 0.23–0.97, p = 0.041) (Table 7).

**Table 7:** Multiple regression analysis of factors associated with successful TB treatment outcome among HIV/TB co-infected patients in Fako Division, 2010.

Variables	Category	TB treatment outcome		Total	AOR	95% CI	p-value
		Unsuccessful No (%)	Successful No (%)				
**Sex**	Male	117(26.0)	333(74.0)	450	1.00	-	-
	Female	106(17.9)	485(82.1)	591	1.64	1.21–2.22	0.001
**Year of TB diagnosis**	2010	36(29.3)	87(70.7)	123	1.00	-	-
	2011	33(26.0)	94(74.0)	127	1.20	0.68–2.11	0.531
	2012	26(23.2)	86(76.8)	112	1.40	0.77–2.55	0.270
	2013	34(21.8)	122(78.2)	156	1.43	0.82–2.48	0.207
	2014	24(17.1)	116(82.9)	140	1.90	1.04–3.45	0.036
	2015	23(14.2)	139(85.8)	162	2.43	1.33–4.43	0.004
	2016	28(20.9)	106(79.1)	134	1.49	0.83–2.66	0.183
	2017	19(21.8)	68(78.2)	87	1.37	0.71–2.63	0.353
TB category	New	195(20.8)	743(79.2)	938	1.00	-	-
	Relapse	13(36.1)	23(63.9)	36	0.48	0.23–0.97	0.041
	Treatment failure	1(12.5)	7(87.5)	8	2.15	0.25–18.38	0.483
	Retreatment	4(28.6)	10(71.4)	14	0.74	0.22–2.50	0.633
	Transferred in	10(22.2)	35(77.8)	45	0.93	0.45–1.95	0.848
ART regimen at TB diagnosis	Yes	185(20.0)	740(80.0)	925	0.64	0.39–1.03	0.067
	No	38(32.8)	78(67.2)	116	1.00	-	-
Cotrimoxazole treatment at TB diagnosis	Yes	205(20.7)	784(79.3)	989	0.65	0.33–1.28	0.216
	No	18(34.6)	34(65.4)	52	1.00	-	-

AOR = Adjusted Odds Ratio; CI = Confidence Interval

## Discussion

Tuberculosis is a common opportunistic infection in people living with HIV/AIDS; both infections are considered major public health problems in sub-Saharan Africa. The study found an HIV-TB co-infection rate of 35.6% and it was higher than that of other previous studies in Ethiopia [5–7], India [8] and Brazil [9], but lower than that recorded in Nigeria [10].

The overall successful TB treatment outcome in this study was 78.6%. Our result is higher than that of previous studies conducted in Africa (Ethiopia 28.9% [11], 30.3% [12], 70.8% [13], Nigeria 48.8% [10], Ghana 64.0% [14] and Cameroon 60.8% [15]) and elsewhere (Malaysia 53.4% [16]). This dissimilarity might be due to the difference in number of study participants involved in the various studies. The rates of death and lost to follow-up may also affect the success of TB treatment. This is evidenced by the differences in rates of death and lost to follow-up observed in our findings compared to the other studies. The rate of successful TB treatment outcome recorded in the present study (78.6%) was nearly in agreement with studies conducted in India 75.0% [17] and 80.0% [18], Vietnam 74.0% [19], Zambia 80.0% [20] and Ethiopia 80.5% [21]. Other studies conducted in India 84.17% [22] and South Africa 82.2% [23] have recorded higher successful TB treatment outcome. However, our finding and those of these others studies are lower than the target of successful TB treatment rate (≥90%) recommended by the WHO [1]. It is worth noticeable and encouraging that a health facility (Baptist Hospital Mutengene) in the South West Region of Cameroon recorded a successful TB treatment out of 94.97% between 2011 and 2012, a success rate higher than the recent WHO recommendation [24].

The availability of facilities for the diagnosis of TB in PLWHA and the increased awareness on the importance of adhering to treatment are contributing factors to TB treatment successes. However, the poor living and feeding conditions of patients in our setting may contribute to the failure in achieving the WHO target, a situation similar for many countries that are failing to achieve adequate successful TB treatment outcomes.

Being a female was found to be significantly associated with a successful TB treatment outcome in our study. The possible explanation for this observation is that women are more willing to follow their treatment compared to men in our socio-economic setting.

In this study, there was no significant difference in TB treatment outcome during the 2010 to 2014 Cameroon national tuberculosis strategic plan compared to the outcomes recorded between 2015 and 2017. However, patients who were diagnosed and treated for TB in in the years 2014 and 2015 showed significantly associated successful TB treatment outcome compared to those who were being treated within 2010–2013. By 2014, the programmes of the millennium development goals were getting to an end, paving ways for an intensified fight of these diseases. Furthermore, the 10 years national programme for the fight against HIV/AIDS and TB in Cameroon were closing in 2014, giving more strength to enable the fight of these diseases. More effort on HIV management can indirectly influence TB treatment outcome among co-infected patients.

In our study, PLWHA who were receiving antiretroviral therapy at the time of TB diagnosis were not significantly likely to have successful TB treatment outcome. Patients on antiretroviral therapy might have been diagnosed late in the course of their HIV disease to have any beneficial effect from the treatment services.

Further, patients who were on Cotrimoxazole prophylaxis were not found to be significantly associated with the successful TB treatment outcome. Cotrimoxazole prophylaxis aids in the prevention of other opportunistic infections. However, it did not lower the chances of an unsuccessful TB treatment among HIV patients.

The major limitation of this review is the use of retrospective secondary data, which is totally limited to whatever was documented in the TB registers in the hospitals. Variables such as treatment adherence, other disease conditions as well as behavioral and social factors which might affect outcome were not routinely captured in patients’ file. In addition, many patients were transferred to other health facilities where it was difficult to ascertain their final treatment outcome.

## Conclusion

This study revealed that successful TB treatment outcome among PLWHA was high, even thoughit was below the target set by the WHO. Being a female, diagnosed and treated for TB in 2014 and 2015, receiving ART and Cotrimoxazole prophylaxis were found to be factors associated with successful TB treatment outcome. We recommend the strengthening of health systems to facilitate a consistent availability of antiretroviral therapy, DOTs and Cotrimoxazole to all HIV and TB co-infected patients.

## Supporting information

S1 File (XLSX)

## Abbreviations

AIDS: Acquired Immunodeficiency Syndrome
ART: Antiretroviral Therapy
DOTs: Directly Observed Short Course
HIV: Human ImmuneDeficiency Virus
SD: Standard deviation
SPSS: Statistical Package for Social Sciences
TB: Tuberculosis
WHO: World Health Organization
